# Supplementary material for: Identifying maintenance hosts for infection with Dichelobacter nodosus in free-ranging wild ruminants in Switzerland: A prevalence study
Source: PLoS One. 2020 Jan 9;15(1):e0219805. doi: 10.1371/journal.pone.0219805 (PMC6952115; doi:10.1371/journal.pone.0219805)
Supplement: S1 Table — Estimated true**/apparent* prevalence of D. nodosus, with corresponding 95% confidence intervals indicated in parentheses and number of tested animals. (PDF) [file pone.0219805.s001.pdf]

Supporting information

S1 Table. Cantons with PCR positive animals. Estimated true**/apparent* prevalence of *D. nodosus*, with corresponding 95% confidence intervals indicated in parentheses and number of animals (positive/tested).

Species								
	Ibex		Red deer		Roe deer		Chamois	
Cantons	Benign	Virulent	Benign	Virulent	Benign	Virulent	Benign	Virulent
UR	0.00% (0.00-23.09) 0/14*	0.00% (0.00-23.09) 0/14*	0.00% (0.00-31.61) 0/9*	0.00% (0.00-31.61) 0/9*	0.00% (0.00-52.71) 0/4*	0.00% (0.00-52.71) 0/4*	6.25% (0.31-30.04) 1/16*	0.00% (0.00-20.12) 0/16*
BE	7.14% (0.36-31.22) 1/14*	0.00% (0.00-23.09) 0/14*	6.25% (0.31-30.04) 1/16*	0.00% (0.00-20.12) 0 /16*	0.00% (0.00-4.51) 0/79*	0.00% (0.00-4.51) 0/79*	0.00% (0.00-13.30) 0/24*	0.00% (0.00-13.30) 0/24*
GR	0.31% (0.00-1.63) 1/319*	0.31% (0.00-1.63) 1/319*	12.36% (7.39-18.82) 23/172**	0.00% (0.00-2.07) 0/172*	0.00% (0.00-7.75) 0/41*	0.00% (0.00-7.75) 0/41*	0.00% (0.00-2.61) 0/136*	0.00% (0.00-2.61) 0/136*
LU	0.00% (0.00-63.15) 0/3*	0.00% (0.00-63.15) 0/3*	0.00% (0.00-40.61) 0/6*	0.00% (0.00-40.61) 0/6*	3.12% (0.15-16.16) 1/32*	0.00% (0.00-9.94) 0/32*	0.00% (0.00-22.51) 0/13*	0.00% (0.00-22.51) 0/13*
VD	9.09% (0.45-40.10) 1/11**	0.00% (0.00-25.63) 0/3*	0.00% (0.00-63.15) 0/3*	0.00% (0.00-63.15) 0/3*	0.00% (0.00-21.50) 0/15*	0.00% (0.00-21.50) 0/15*	0.00% (0.00-30.61) 0/6*	0.00% (0.00-30.61) 0/6*
VS	0.00% (0.00-2.67) 0/133*	0.00% (0.00-2.67) 0/133*	6.99% (1.96-16.49) 7/83**	0.00% (0.00-4.29) 0/83*	0.00% (0.00-18.9) 0/17*	0.00% (0.00-18.9) 0/17*	0.00% (0.00-3.20) 0/111*	0.00% (0.00-3.20) 0/111*
